# Genistein as a regulator of signaling pathways and microRNAs in different types of cancers

**DOI:** 10.1186/s12935-021-02091-8

**Published:** 2021-07-21

**Authors:** Zeeshan Javed, Khushbukhat Khan, Jesús Herrera-Bravo, Sajid Naeem, Muhammad Javed Iqbal, Haleema Sadia, Qamar Raza Qadri, Shahid Raza, Asma Irshad, Ali Akbar, Željko Reiner, Ahmed Al-Harrasi, Ahmed Al-Rawahi, Dinara Satmbekova, Monica Butnariu, Iulia Cristina Bagiu, Radu Vasile Bagiu, Javad Sharifi-Rad

**Affiliations:** 1 Office of Research Innovation and Commercialization (ORIC), Lahore Garrison University, Sector-C, DHA Phase-VI, Lahore, Pakistan; 2 Department of Healthcare Biotechnology, Atta-ur-Rahman School of Applied Biosciences (ASAB), National University of Sciences and Technology (NUST), Islamabad, 44000 Pakistan; 3 Departamento de Ciencias Básicas, Facultad de Ciencias, Universidad Santo Tomas, Santiago, Chile; 4 Center of Molecular Biology and Pharmacogenetics, Scientific and Technological Bioresource Nucleus, Universidad de La Frontera, 4811230 Temuco, Chile; 5 School of Life Sciences, Lanzhuo University, Lanzhou, 730000 People’s Republic of China; 6 Department of Biotechnology, Faculty of Sciences, University of Sialkot, Sialkot, Pakistan; 7 Department of Biotechnology, BUITEMS, Quetta, Pakistan; 8 Institute of Biochemistry and Biotechnology, University of Veterinary and Animal Sciences, Lahore, Punjab Pakistan; 9 Department of Life Sciences, University of Management Sciences, Lahore, Pakistan; 10 Department of Microbiology, University of Balochistan, Quetta, Pakistan; 11 Department of Internal Medicine, University Hospital Centre Zagreb, School of Medicine, University of Zagreb, Zagreb, Croatia; 12 Natural and Medical Sciences Research Centre, University of Nizwa, Birkat Almouz, Nizwa, 616 Oman; 13 High School of Medicine, Al-Farabi Kazakh National University, Almaty, Kazakhstan; 14 Banat’s University of Agricultural Sciences and Veterinary Medicine “King Michael I of Romania” From Timisoara, Timisoara, Romania; 15 Victor Babes University of Medicine and Pharmacy of Timisoara Discipline of Microbiology, Timisoara, Romania; 16 Multidisciplinary Research Center on Antimicrobial Resistance, Timisoara, Romania; 17 Preventive Medicine Study Center, Timisoara, Romania; 18 Phytochemistry Research Center, Shahid Beheshti University of Medical Sciences, Tehran, Iran

**Keywords:** Genistein, miRNAs, Nano-formulations, Signaling pathways, Therapeutics

## Abstract

Cancers are complex diseases orchestrated by a plethora of extrinsic and intrinsic factors. Research spanning over several decades has provided better understanding of complex molecular interactions responsible for the multifaceted nature of cancer. Recent advances in the field of next generation sequencing and functional genomics have brought us closer towards unravelling the complexities of tumor microenvironment (tumor heterogeneity) and deregulated signaling cascades responsible for proliferation and survival of tumor cells. Phytochemicals have begun to emerge as potent beneficial substances aimed to target deregulated signaling pathways. Isoflavonoid genistein is an essential phytochemical involved in regulation of key biological processes including those in different types of cancer. Emerging preclinical evidence have shown its anti-cancer, anti-inflammatory and anti-oxidant properties. Testing of this substance is in various phases of clinical trials. Comprehensive preclinical and clinical trials data is providing insight on genistein as a modulator of various signaling pathways both at transcription and translation levels. In this review we have explained the mechanistic regulation of several key cellular pathways by genistein. We have also addressed in detail various microRNAs regulated by genistein in different types of cancer. Moreover, application of nano-formulations to increase the efficiency of genistein is also discussed. Understanding the pleiotropic potential of genistein to regulate key cellular pathways and development of efficient drug delivery system will bring us a step towards designing better chemotherapeutics.

## Introduction

Genetic mapping and genome wide analysis have enabled researchers to pinpoint different genetic variants of cell signaling cascades. Advancements in functional genomics have laid foundation of a new frontier in molecular oncology and understanding of molecular mechanisms involved in genetic variants [1, 2]. Genistein is an isoflavone which could be found in soybean, chickpeas, and another soy-based foodstuff. It has a broad range of anti-cancer properties. Genistein has been analyzed and reviewed in a number of papers for its anti-cancer effects [3, 4]. However, in this review we have focused on the genistein mediated modulation of signaling cascades in various types of cancer. For the search of literature, PubMed and google scholar was explored. Input in the form of keywords: “genistein and cancer”, “genistein and cell pathway”, “genistein nanoformulations” and “genistein clinical trials” was loaded in the search bars of selected databases and studies relevant to the aims and objectives of the current review are included. This multi-component review is divided into several sections. The first section is dealing with molecular interactions of genistein and its role in different cellular pathways.

### Molecular interactions of genistein and its role in different cellular pathways

**Fig. 1:**
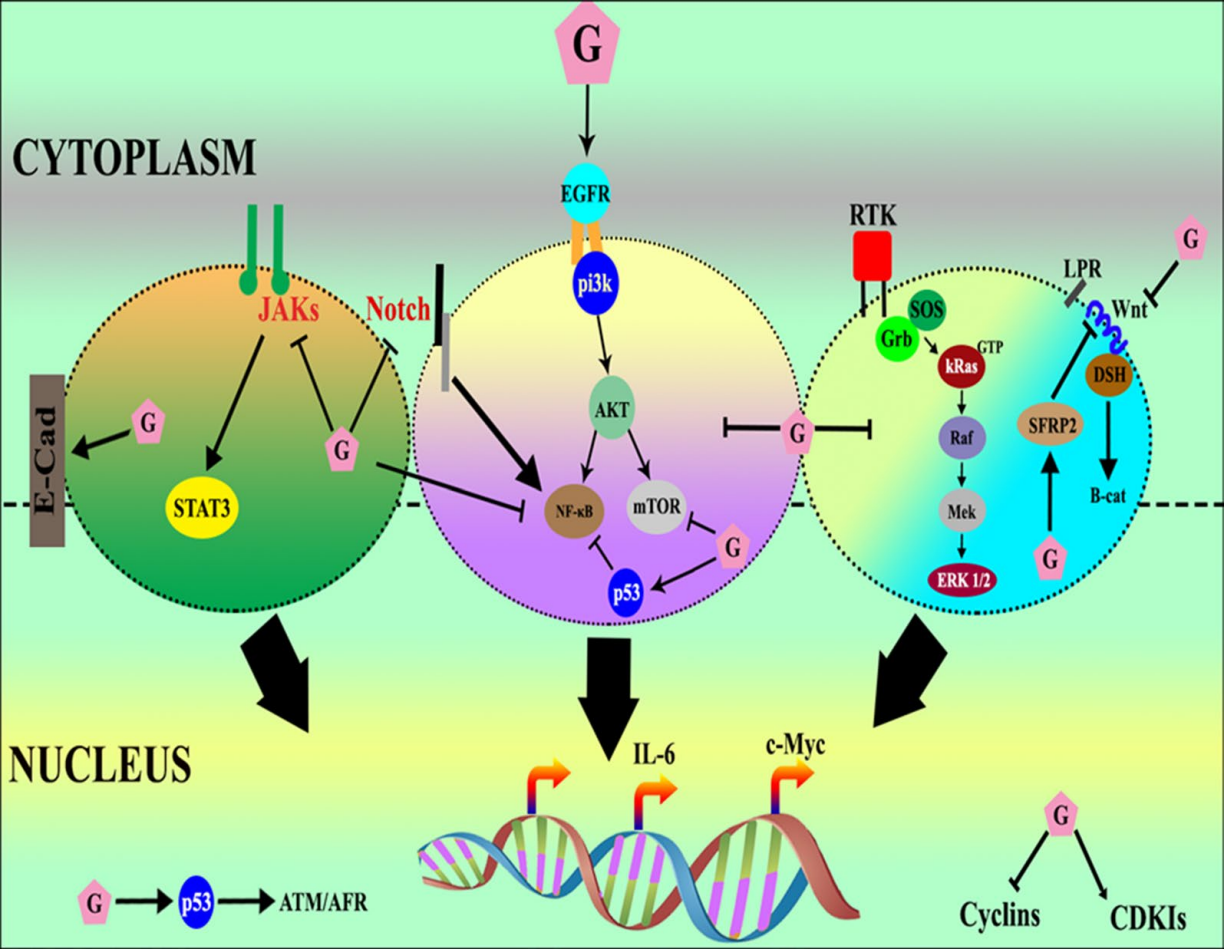
Regulatory role of genistein in cellular pathways. Genistein modulates receptor tyrosine kinase (RTKs) signal transduction. It prevents the activation of nuclear factor kappa-light-chain-enhancer of activated B cells (NF-κB) explicitly either by the Akt pathway, Notch pathway, or by P53. It modulates the cell cycle by inhibiting cell cycle kinases and up-regulating Cyclin-Dependent Kinase inhibitors (CDKIs). It regulates ataxia telangiectasia mutated/F-box protein (ATM/AFR) through p53. Genistein also modulates metabolic pathways such as gluconeogenesis via the mTOR pathway. It up-regulates the Wnt pathway inhibitors and suppresses signal transduction by the Wnt pathway by inhibiting frizzled receptor and low density lipoprotein receptor-related protein (LPR) interactions and reduces β-catenin (B-cat) cytoplasmic accumulation. Genistein in the figure is depicted by “G’

The medical benefits of genistein are well documented. Several studies have been performed to better understand the mechanisms of its beneficial effects in different diseases. In vivo and in vitro analyses indicated that genistein can modulate numerous signaling cascades in several types of cancer cells, that results in decreased cell viability, and promotion of cell death [5]. Figure 1 illustrates the molecular interactions of genistein in different cellular pathways.

### EGFR/Akt/NF-κB pathway modulation

Genistein directly suppresses the activity of Akt and therefore this substance promotes the inactivation of its downstream signaling pathways, including nuclear factor kappa-light-chain-enhancer of activated B cells (NF-κB) [5–7]. The electrophoretic mobility shift assay further demonstrated that treatment with genistein directly inactivates NF-κB in MDA-MB-231 cells [6]. Genistein also inhibits Akt activation by preventing its upstream EGF signal activation [6, 7]. It can be concluded that genistein inhibits NF-κB activation either by EGF and Akt inactivation or by directly inactivating it.

### Notch/NF-κB pathway modulation

Genistein modulates several other pathways that eventually prevents the activation of NF-κB. It decreases the expression of Notch1, which consequently suppresses the activation of NF-κB [8, 9]. Notch1 expression is modulated in healthy cells by tumor suppressor miR-34a. However, its downregulation in cancer cells promotes Notch1 signaling. Xia et al. demonstrated that genistein also enhances miR-34a expression, which consequently targets Notch1 [10]. Targeting the Notch pathway by genistein has been also reported in colon cancer by Zhou et al. [11]. Downstream targets of Notch1/NF-κB pathway were also demonstrated. According to these authors, genistein suppresses colon cancer cell migratory potential by targeting Notch 1 and disrupts phosphorylation of NF-κB and by up-regulating E-cadherin. Genistein also normalizes the metalloproteinases/tissue inhibitors of metalloproteinases (MMPs/TIMP) concentration balance by Notch2/Jagged1 pathway in the cells [12]. Signal transduction by the NF-κB pathway is also attenuated by genistein mediated p53 production. P53 increased expression disrupts the subcellular localization of NF-κB and stops it in the cytoplasm. This affects its DNA binding capability and hinders transcription of cytokines genes such as *IL6*. Therefore, genistein treatment decreases transcription and translation of cytokines by inhibiting NF-κB in LPS-stimulated human monocyte-derived dendritic cells [13].

### JAK-STAT/NF-κB pathway modulation

Genistein targets JAK/STAT signaling cascade components and prevents activation and nuclear translocation of NF-κB [14]. A study demonstrated that genistein specifically interacts and suppresses IL-6 receptor-associated JAK2. Its particular targeting of STAT3 in rheumatoid arthritis was also reported [15]. IL-6 receptor is a type-I cytokine receptor. It would be interesting to investigate the effects of genistein on other types of cytokine receptors. Furthermore, it would be important to perform studies analyzing the regulatory role of genistein on cytokine/cytokine receptor interaction.

### JAK/RAS/RAF pathway modulation

Genistein targets the activation of JAKs by preventing its phosphorylation which, consequently, suppresses the activity of downstream pathways. JAKs induce replacement of RAS-GDP with RAS-GTP that turns on RAS by adaptor proteins Grb2 and SOS. Activated RAS then phosphorylates RAF (MAPKKK) that eventually causes ERK1/2 activation and the expression of associated genes such as c-Myc, and c-Jun. Genistein targets JAKs phosphorylation and, on the other hand, it prevents ERK activation that inhibits eventually the expression of transcription factors Myc and Jun [16, 17]. Genistein is also evidenced to directly inhibit phosphorylation activation of ERK and STAT3 [18].

### Akt/mTOR pathway modulation

Several cellular pathways get activated upon activation of Akt, which cumulatively divert cells’ trend from apoptosis to cell survival. Activation of the NF-κB pathway puts a temporary hold on apoptosis and promotes cytokines release, which further increases the cellular communication. Similarly, mTOR pathway activation by Akt allows the cell to regulate metabolism and divide and proliferate. A study of endometrial cancer cells showed anti-cancer effects of genistein, concerning its capability to attenuate phosphorylation of mTOR pathway components [19]. In a similar study, a concomitant increase in progesterone receptor transcription and a decrease in estrogen receptor transcription were also reported, because of treatment with genistein and novasoy. In acetaminophen (APAP)-induced liver injury in rats the treatment with genistein improved the injury by up-regulating Silent information regulator 1 (SIRT1; a negative regulator of mTOR pathway). Enhanced SIRT1 expression activated Nrf2 signaling that improved the injury-induced oxidative stress [20]. Treatment with genistein also reduced oxidative stress during neuronal injury by modulation of Nrf2 signaling [21, 22]. Further, genistein treatment also enhanced SIRT1 expression in schistosomiasis-induced hepatic fibrosis that led to protective effect via α-SMA and TGF-β expression down-regulation [23]. Genistein-induced SIRT1 expression also down regulates osteopontin expression [3]. However, no study has been performed so far to investigate the role of genistein on regulators of mTOR and Nrf2 pathways in different types of cancer. Better understanding of the effects of genistein on these pathways on molecular level will further help in understanding the mechanisms by which this flavonoid achieves its possible anti-tumor effects.

### Canonical Wnt pathway modulation

Wnt signaling pathway modulation by treatment with genistein has also been documented. Genistein takes the advantage of a tool of epigenetic modification—DNA methylation to modulate the expression of several Wnt pathway associated genes [24, 25]. As showed by bisulfate genomic sequencing and methylation-specific PCR, the treatment with genistein suppresses the methylation of CpG islands on the promoter region of Wnt5a in colon cancer cell lines DLD-1, SW480, and SW1116 [26]. Immunoblotting analysis and RT PCR analysis of colon cancer cell lines treated with genistein have shown that genistein reduced the nuclear concentration of β-catenin and enhanced its phosphorylation [27]. Treatment with genistein also enhanced β-catenin phosphorylation in mammary cell lines, restricting it to the cytosol and down-regulated Wnt1 signaling and its associated genes such as cyclinD1 and cMyc [28]. Genistein also attenuates Wnt signaling by up regulating the Wnt pathway's antagonists FRP2 in a human colon cancer cell line indicating the modulatory role of genistein on the Wnt pathway [27]. Another study [29] reported that genistein down-regulates an onco-miRNA-miR-1260b which targets sFRP2 in colon cancer cells. Different than sFRP2, luciferase activity assay also indicated Dkk2 and Smad4 as molecular targets of miR-1260b, suggesting that genistein might have potential regulatory activity on TGF signaling as well. However, no experimental evidence exists to confirm the activity of genistein against TGF signaling. Genistein also promotes dose dependent up-regulation of WIF1which is an inhibitor of Wnt [30].

### Estrogen receptor and associated VEGFR modulation

Genistein also regulates the activity of VEGFR receptor. Kinase activity analysis and immunoprecipitation study have indicated that phosphorylation status of VEGFR and its tyrosine kinase activity were reduced after treatment with genistein [31]. Genistein also targets estrogen receptor expression and suppresses the pathways associated with it. The activation of estrogen receptor also promotes VEGFR-2 expression. Treatment with genistein inhibits estrogen receptor and the expression of estrogen receptor-induced VEGFR [32, 33]. Together with enterolactone genistein also inhibited estradiol-mediated VEGFR-2 expression [34].

### Genistein-mediated cell cycle regulation leading to apoptosis

Genistein also causes G2/M phase arrest [35]. It interacts on molecular level with several cell cycle regulators and cell cycle progression-associated proteins and inhibits cancer cell growth [36]. It regulates the members of kinesins in protein family, particularly KIF20, and attenuates gastric cancer cell viability by accumulating cells in the G2/M phase [37]. Western blot analysis further helped to explain the mechanism of action of genistein and suggested that genistein can block the translation of Cdc25C, CDK1, cyclinA, and cyclinB1 and up-regulate the expression of CDK inhibitor, p21WAF1/CIP1 in MDA-MB-231 and T24 cells [35, 38]. In kidney cancer, genistein up-regulates the expression of another CDK inhibitor-CDKN2a, by inducing its epigenetic modification in a time and concentration dependent manner [39].

Genistein also stabilizes the activation and phosphorylation of MAPK by modulating the RAS/RAF signaling pathway [19, 35]. Genistein is also reported to inhibit two vital cell survival pathways—Akt pathway and JAK/STAT pathway. Genistein disrupted JAK1/2 and Akt phosphorylation, which consequently, prevented the phosphorylation activation of their downstream targets STAT3 and MDM2 and restricted their localization to the cytoplasm. MDM2 inactivation reciprocally allowed p53 to show its tumor suppressor effects by mediating activation of ATR, ATM, H2AX, and CHK2, leading to cell senescence [40]. Genistein gradually promotes cell death by the intrinsic pathway. It causes proteolytic cleavage dependent activation of PARP as well as activator and executioner caspases of the intrinsic pathway. It also causes aberration in Bcl2/Bax, leading to the disruption of the mitochondrial membrane and production of ROS [38, 40, 41]. An in vitro study demonstrated that long-term exposure to genistein significantly reduced cellular levels of the EGF receptor, which is upstream of the Akt and JAK/STAT pathway [40]. It could be concluded that genistein inactivates EGFR which inhibits phosphorylation of its downstream molecules such as JAK and Akt, leading to cell senescence and apoptosis by p53.

Genistein interacts with several molecular pathways in different diseases and can be used as a therapeutic substance. However, several aspects of its mechanism of action are yet to be explored. For instance, genistein can modulate the glucose pathway and promote secretion of insulin. Therefore, its activity in inducing the Weber effect in cancer can be investigated. Its role in modulating the Nrf2 pathway in cancer cells can be also investigated. This can provide a better insight in crosstalk between cells' metabolic pathways and inflammatory pathways in cancer.

### Cancer cell specific cytotoxic influence of genistein

Evidence indicates the cancer cell specific cytotoxic effect of genistein. Its treatment does not influence the viability and growth of normal cells. In human leukemia HL-60 cells, genistein along with bleomycin exposure for three hours promoted micro nucleation and DNA damage while its treatment for same duration in normal lymphocytes reduced micronucleation and DNA damage [42]. Similarly, comparative to breast cancer malignant cells (MDA-MB-231), genistein treatment down-regulated p21WAF1 expression in normal breast epithelial cells (MCF10A and MCF12A), suggesting cancer cell specific cytotoxic effect of genistein [43]. In hepatic cellular carcinoma HepG2, Huh7 and Hep3B cell lines, pro-apoptotic influence of genistein was reported to be more pronounced than normal hepatic L-02 cells [44]. Likewise, chitosan-encapsulated genistein treatment in colon cancer HT-129 cells promoted anti-angiogenesis while the viability of normal cells was not effected in tested genistein concentrations [45]. Outcomes of these investigations highlight selective effect of genistein and suggests its therapeutic implementation for curbing human carcinogenicity.

### Regulation of miRNAs by genistein in different types of cancer

Since the discovery of non-coding RNA sequences, their role in almost all cellular processes by regulation of different genes is becoming more and more evident. MicroRNAs (miRNAs) are a special class of non-coding. These non-coding RNAs are short (approximately 18 to 25 nucleotides long) endogenous molecules, which are very important egulators of different cellular processes including those in tumor cells [46]. The exact mechanism of how miRNAs regulate gene expression is complex and still not fully understood. However, what is known so far, they bind to 3’ UTR region of target RNA molecules, destabilize them and deteriorate the process of translation [47]. Recently, it has been found that numerous miRNAs are involved as potential regulators of key players of almost all kinds of cancer. It is very important to explain the role of miRNAs in different tumors since their expressions could be used as diagnostic and prognostic tools. Moreover, miRNAs target various genes involved in cancer and related to cellular processes such as cell proliferation, cell cycle, and apoptosis thus indicating that they might be useful in cancer treatment [48, 49]. One strategy in cancer treatment might be the use of extracts from natural products since they could regulate miRNAs expression and subsequently provide tumor suppression [50]. Some miRNAs which can be regulated by genistein and could play a role in suppression of different tumor cells growth and metastasis will be discussed (Table 1).

**Table 1:** List of genistein modulated miRNA and their targets in different cancers

Cancers	miRNAs	Expression	Targets	Refs.
Breast cancer	miR-155	Down-regulated	FOXO3, PTEN, casein kinase, p27	[51]
	miR-23b	Up-regulated	PAK2	[52, 53]
Prostate cancer	miR-151	Down-regulated	CASZ1, IL1RAPL1, SOX17, N4BP1 and ARHGDIA	[54]
	miR-34a	Up-regulated	HOTAIR	[55]
	miR-574-3p	Up-regulated	RAC1, EGFR, EP300	[56]
	miR-1296	Down-regulated	minichromosome maintenance gene family	[57]
	miR-200 miR-141	Up-regulated	DNMT3A and TET1/TET3	[58]
	miR-1260b	Up-regulated	sFRP1 and Smad4	[59]
Colorectal cancer	miR-95	Down-regulated	Akt, SGK1	[60]
Pancreatic cancer	miR-223	Down-regulated	Fbw7	[61]
	miR-34a	Up-regulated	Notch-1	[62]
	miR-27a	Down-regulated	Sprouty2	
Uveal melanoma	miR-27a	Down-regulated	ZBTB10	[63]
Ovarian cancer	miR-27a	Down-regulated	Sprouty2	[64]
Lung cancer	miR-27a	Up-regulated	MET	[65]
	miR-873-5p	Down-regulated	FOXM1	[66]

### Breast cancer

A plethora of evidence has indicated an important role of genistein as an anti-cancerous substance in various types of cancer. It can affect the expression of numerous miRNAs, which control tumor regulation in breast cancer. For instance, genistein can down-regulate miR-155, resulting in reciprocal increase in the expression of miR-155 targets which are FOXO3, PTEN, casein kinase, and p27 in MDA-MB-435 and Hs578t breast cancer cell lines [51]. The target proteins of miR-155 go through complex signaling cascades and act as tumor suppressors. For instance, PTEN down-regulates the phosphatidylinositol-3-kinase (PI3K)/protein kinase B (Akt) signaling, which regulates different cellular processes such as motility, invasion, proliferation and survival [67]. Thus down-regulation of miR-155 indirectly helps in breast cancer suppression. Another microRNA named miR-23b was upregulated for MCF-7 breast cancer cells after genistein treatment indicating that genistein might cause suppression of breast cancer [52]. MiR-23b could regulate cytoskeletal reorganization and contribute to reduced cell motility and invasion via its target PAK2 in breast cancer cell lines [68].

### Prostate cancer

Genistein can also down-regulate miR-151 in PC3 and DU145 cells and contribute to inhibition of cell migration and invasion in prostate cancer by affecting the expression of several miR-151 target proteins such as CASZ1, IL1RAPL1, SOX17, N4BP1 and ARHGDIA as determined by computational bioinformatics and 3′ luciferase reporter assays [54]. As a response to decreased miR151, CASZ1 expression increases that contributes to inhibition of tumor migration in vitro and suppresses tumor genesis in vivo [69]. miR-34a targets oncogenic HOTAIR and decreases its expression resulting in suppression of prostate cancer in PC3 and DU145 PCa cell lines [55]. Another study reported the up-regulation of miR-574-3p in prostate cancer and subsequent suppression of tumor growth. The results were consistent even with transient expression of miR-574-3p. Many important players of oncogenic pathways such as RAC1, EGFR and EP300 are targeted and down-regulated by miR-574-3p [56]. Mini-chromosome maintenance (MCM) gene family plays an important role in DNA replication and is often associated with various types of cancer. Majid et al. reported the up-regulation of MCM2 and down-regulation of miR-1296 in prostate cancer cells. They have also found that exposure to genistein down-regulates MCM2 and also up-regulates miR-1296, resulting in tumor suppression [57]. These studies clearly show that genistein could suppress prostate cancer by interacting with many miRNAs and subsequently regulating their targets.

However, the exact mechanism of these effects of genistein is still not fully understood. A study showed down-regulation of miR-200 and miR-141 in prostate cancer cells. This down-regulation of both miRNAs was governed by inversely correlated methylation status of promoter regions of these miRNAs. Genistein treatment resulted in demethylation of the promoter CpG sites near miR-200c and miR-141 loci, as well as increased expression and subsequently suppression of prostate cancer [58]. In prostate cancer cells, the expression of sFRP1 and Smad4 are down-regulated by increased expression of miR-1260b, resulting in cell proliferation, invasion, migration and TCF reporter activity. Genistein treatment in prostate cancer significantly down-regulated miR-1260b and caused subsequent up-regulation of its targets sFRP1 and Smad4 by DNA demethylation and histone modifications, which caused a suppression of prostate cancer cells [59].

### Colorectal cancer

Genistein proved to be effective in suppressing colorectal cancer cells by down-regulation of miR-95. The expression of Akt was downregulated, which was attributed to inhibit the phosphorylation at T308 within catalytic domain of Akt and its activation which might play a role in apoptosis. Genistein could also suppress xenograft tumors in mouse tissues [60]. Several other miRNAs could directly or indirectly have effect on the expression of Akt and SGK1. Nevertheless, their role in colorectal cancer suppression still needs to be further explored.

### Pancreatic cancer

Genistein can inhibit oncogenic miR-223 expression resulting in cell growth inhibition and apoptosis induction in pancreatic cancer cells. Down-regulation of miR-223 results in up-regulation of its target Fbw7 [61]. Fbw7 is an E3 ligase which acts as a tumor suppressor. Its substrate SHOC2 is a RAS activator and phosphorylation of SHOC2 at Thr507 facilitates binding of Fbw7. Consequently, SHOC2 is degraded which terminates RAS-MAPK signals and inhibits cell proliferation [70]. Genistein treatment also significantly increases miR-34a expression and subsequently, induces down-regulation of Notch-1, resulting in cell growth inhibition and induction of apoptosis [62]. Another study showed that treatment with genistein could result in decreased expression of miR-27a, which caused suppression of pancreatic cancer [71].

### Other types of cancer

A single miRNA can interact with several proteins thus regulating different signaling pathways as well as having effects on different tumors. For example, Sun et al. found a link between genistein and miR-27a and reported the inhibition of uveal melanoma cell growth as a response to treatment with genistein [63]. Later, a number of studies analyzed the effects of genistein on oncogenic miR-27 which is responsible for different tumors control. Genistein down-regulated miR-27 expression in SKOV3 cells which resulted in increased expression of a miR-27 target proteinSprouty2 and suppression of ovarian cancer growth and migration [64]. As mentioned earlier, treatment with genistein also resulted in regulating miR-27a in pancreatic cancer cells [71]. Another study showed that activation of miR-27a occurred as a response to genistein treatment due to mediating MET signals in lung cancer cells [65].

### Nano-formulations of genistein

Poor water solubility, non-specific targeting, low serum availability, rapid metabolism and excretion are the major obstacles influencing the clinical effectiveness of genistein [72]. A suitable drug delivery system seems to be an important strategy to overcome the hinderances posed in its clinical implementation. Recent research has led to the development of robust formulations of genistein that could resolve the issues of limited bioavailability, solubility and specific targeting [73]. Particulate drug carriers such as micro particles and colloidal system have gained a lot of attention. Particulate drug carrier systems, for instance solid-lipid-particulate system (SLPs), can increase the solubility, stability and control bioavailability of the drug, along with reducing the cytotoxicity caused by synthetic materials used in encapsulation. Particle size in SLPs carrier system is of great significance as the diffusion rate and release of the carried drug directly depends upon it that could influence drug’s oral bioavailability [74, 75]. The use of SLPs as carrier for delivery of genistein has been proven to be effective in both in vitro and in vivo studies [76]. Pharmacokinetics study showed that SLPs increased the bioavailability of the genistein by slowing its dissolution rate. Furthermore, it was proven that particle size of the SLPs was the most important factor that affected the drug release in SD rat model. It was concluded that SLPs loaded with genistein, and solid lipid nanoparticles enhanced the bioavailability when compared with genistein suspension. Oral route of drug delivery increases the drug availability [77].

Oral drug administration is the most convenient form of drug delivery for patients. Oral delivery also allows controlled release of target drugs. Such formulations contain a range of ingredients that facilitate target specific delivery of phytochemicals [78]. Recently many efforts were made towards the development of isoflavone tablets for oral drug administration. Compact formulations of tablet based genistein and diadzein have been developed with various disintegrating substances to determine their specificity. Several studies have shown that the formulation of soy extracts prepared by direct compression method containing genistein and daidzein (50% w/w) increased cohesiveness and flow rates of oral tablets [79]. Polymeric micelles have been employed in pharmaceuticals, drug delivery and gene delivery systems. They have characteristic hydrophobic core surrounded by hydrophilic part that facilitates dispersion and provides stability of target drug release. Limited data is available regarding the utilization of polymeric micelles as tool for the delivery of genistein in cancer. A study has demonstrated that pluronic F127 polymeric micelle prepared by solid dispersion method containing genistein efficiently increased bioavailability and solubility when administrated orally to rats when compared to genistein powder [80]. These findings indicated that polymeric micelles might be a potent drug delivery system for oral application of genistein.

One of the most innovative and non-invasive approaches for drug delivery is the use of nanoparticles (NPs). Their size, surface properties and release of pharmacologically active substances make them suitable candidates for enhancing therapeutic efficacy and optimal dose regimen [81]. The key characteristics of nanoparticle-based drug delivery system are that the drug can be dissolved, entrapped, encapsulated, and attached to nanoparticle matrix. Therefore, NPs have a lot of potential to be implemented in therapeutics. A number of studies have been performed which have explored the therapeutic efficacy of NPs based drug delivery platforms for the treatment of cancer [81–83]. Nanometric porous metal–organic frameworks (nanoMOFs) are perceived as an appealing system for the delivery of drugs. Mesoporous MIL-100(Fe), a biocompatible, easy to synthesize and high porous NP, have been evaluated for its efficacy for encapsulating genistein. Approximately 28% (wt) of genistein was loaded in MIL-100 with simple impregnation method. Computation analysis estimated its sustained release for three days while genistein loaded MIL-100, orally administered in mice model, revealed its highly improved bioavailability [84]. Genistein in combination with Eudragit using a NPs system increased the oral bioavailability of genistein. This formulation consisted of an internal organic phase containing genistein and Eudragit and an external aqueous phase with a 1% poloxamer-188 as surfactant. Surfactant significantly increased the stability of the nano-delivery system. In contrast to genistein capsule, the Eudragit nano-formulation prolonged the oral delivery of genistein in vivo [85]. All these findings suggest that using NPs system for genistein application can resolve the problems of bioavailability and stability of this substance. Genistein incorporation into PEGylated Silica hybrid nanomaterials (GEN-PEG-SiHNM) reduced cellular growth in HT29 human colon cancer cell lines via modulation of endogenous anti-oxidant enzymes such as hydrogen peroxide. This had a direct effect on triggering autophagy and apoptosis. These findings indicate that GEN-PEG-SiHNM could be implemented as potential therapeutic approach for colorectal cancer in near future [86]. A study has demonstrated that TPGS-b-PCL nanoparticles loaded with genistein effectively reduced tumor cell growth both in vivo and in vitro. PGS-b-PCL copolymer was synthesized from ε-caprolactone initiated by d-α-tocopheryl polyethylene glycol 1000 succinate (TPGS) which enabled the formation of TPGS-b-PCL nanoparticles by ring opening polymerization. TPGS-b-PCL nanoparticles loaded with genistein did not only enhance bioavailability of genistein when compared with PCL (pristine-Genistein loaded NPs), but also had more cytotoxicity and other anti-proliferative characteristics as demonstrated by MTT and colony formation experiments. TPGS-b-PCL nanoparticles were found to be effective in suppressing tumor growth in HeLa xenograft tumor model of BALB/c nude mice. These findings suggest that TPGS-b-PCL nanoparticles could enhance anti-tumor effects both in vitro and in vivo [87]. A study demonstrated that folic acid conjugated chitosan nanoparticles carrying genistein (FGCN) were efficacious in reducing tumor growth in HeLa cell lines when compared with genistein. The FGCN also had a superior cytotoxic effect. It has been demonstrated that use of folate delivery system could enhance anti-tumor efficacy in cervical cancer [88]. A combination of genistein loaded doxorubicin polypeptide nanoparticles (DOX-NPs) improved the adverse effects caused by doxorubicin-HCl and reduced the production of ROS in prostate cancer cells. DOX-NPs facilitated the down-regulation of apurinic/apyrimidinic endonuclease 1 (APE1)—an enzyme important for oxidative DNA repair. APE1 expression up-regulation enhances the ability of cancer cells to recognize DNA damage under oxidative stress and promotes DNA damage repair. Thus DOX-NPs treatment halted cancer cells’ capability to repair DNA damage that reduced the production of ROS and DNA repair in prostate cancer cells. DOX-NPs also prevented the distant metastases of prostate cancer cells by down-regulation of APE1 and aggressive ROS production in prostate cancer cells [89]. Genistein-miR-29b loaded hybrid nanoparticles (GMLHN) suppress tumor growth in non-small cell lung cancer (NSCLC) cell line A549. The Genistein-miR-29b were loaded in mucin-1 aptamer (MUC-1) functionalized hybrid nanoparticles which successfully inhibited the expression of target oncogenes such as pAkt, MCL-1, DNMT3B and PI3K/Akt in A549 and MRC-5 cells. These finding suggested that GMLHN have a potential to be used as a therapeutic tool for NSCLC and other malignant tumors because of their ability of multiple targeting as well as their ability to efficiently deliver their load [90]. Gold NP-loaded genistein was assessed for its anti-cancer role in prostate cancer cells. Genistein selectivity for prostate cancer and bioavailability was significantly improved by conjugating it with gold NP [91]. However, the chemotherapeutic applications of genistein are influenced by its before mentioned unwanted properties. They can be overcomed by amalgamation of genistein with cross-linked carboxy-methylated chitosan (CMCH) iron oxide nanoparticles. This recently developed delivery system has shown greater biocompatibility (surface to volume ratio, easy absorption, and low immunogenic responses) when compared with macro scale substances. Iron Oxide-CMCH-genistein formulation increased water solubility and apoptosis in SGC-7901 cancer cells when compared to with genistein. These findings suggest that iron oxide-CMCH-genistein nano-formulation can be a multifunctional drug delivery platform for combination therapy and chemotherapy [92]. Genistein is also an epigenetic modulator. Polycomb protein seems to be involved in epigenetic repression of the oral squamous cell carcinoma (OSCC). This suggests poor prognosis and development of aggressive phenotype. Using lactalbumin as a medium to create genistein nanoparticles Dev et al. demonstrated that genistein nanoparticles (GLNPs) efficiently reduced the expression of polycomb protein and impeded the growth of oral squamous cell carcinoma. GLNPs administration enhanced ROS production followed by BAX expression and activation of caspase-3 in JHU011 and L929 fibroblast cell lines of OSCC. GLNPs also directly targeted the EZH2 via suppression of the expression of 3PK and enhanced bioavailability, biocompatibility and distribution of genistein in vivo [93]. Intestinal absorption of genistein was attempted to improve by loading it in solid lipid nanoparticles (SLN). In vitro analysis suggested improved intestinal absorption of SLN-loaded genistein [94]. Genistein complexed with phospholipids has been demonstrated to improve its solubility and enhance its accumulation in hepatic cells. Moreover, conjugated genistein also retained anti-cancer effect against hepatic carcinoma [95].

### Clinical studies evaluating the therapeutic potential of genistein

Based upon promising reports of genistein in preclinical studies this substance has been tested in various clinical trials. Since epidemiological and pre-clinical studies suggested that genistein had anti-proliferative effects in colorectal cancer, its efficacy as a potent anti-cancer drug was evaluated in combination with chemotherapy in metastatic colorectal cancer. In a trial including 13 patients genistein was very efficient in combination with FOLFOX or FOLFOX-bevacizumab. None of these patients had adverse effects. This study confirmed that adding genistein to FOLFOX or FOLFOX–bevacizumab was safe and tolerable but larger clinical trials are needed to confirm the safety of its use [96]. A randomized phase II trial has showed that genistein could reduce the adverse urinary tract symptoms associated with intra-vesical therapy and improved quality of life of patients with bladder cancer. In this trial genistein was administrated together with Bacillus Calmette Guerin intra-vesicle therapy. Genistein also inhibited tumor growth by suppressing the expression of key enzymes involved in cellular growth [97]. Genistein has the potential to modulate the expression of key genes involved in metastasis and apoptosis such as the MALAT1 and BASP1 in prostate cancer. Genistein also reduced the expression of HCF2 gene involved in cell migration. Based upon these findings it has been demonstrated that administration of genistein decreased the expression of HCF2 and thus prevented cell migration of the prostate cancer cells and prevented invasion and metastasis. A clinical trial suggested that prolonged administration of genistein could prevent occurrence of prostate cancer by modulating the expression of MEK4 and MMP-2 [98]. Genistein administration (30 mg) to prostate cancer patients reduced the methylation of several genes (ADCY4, NEU1, CYTSB and RBM28) and reduced the expression of oncogenes such as MYC and PTEN. These findings suggest that genistein could be implemented as a useful substance for the treatment of prostate cancer [99]. A phase II trial study is ongoing aiming to evaluate the role of genistein in reduction of diabetes and cardio-metabolic dysfunction in patients with prostate cancer. Androgen inhibition therapy is currently the most suitable approach for treatment of aggressive prostate cancer. However, androgen inhibition therapy has many adverse effects. One major adverse effect is cardio-metabolic dysfunction. In this phase II trial administration of genistein has been reported to reduce diabetes and cardiometabolic dysfunction [100]. A randomized pilot clinical trial has reported that administration of genistein reduced adverse effects associated with chemotherapy in children with solid tumors and lymphomas. Genistein prevented growth of tumor cells and made them more responsive towards chemotherapy [101].

## Conclusion

Advancements in the field of single cell profiling and transcriptomics have increased the understanding of tumor microenvironment and helped to find new answers to cancer proliferation and metastasis. With techniques becoming more and more sensitive there is a need to conceptually analyze single-cell data at tissue and organ level. Thanks to the next generation of sequencing and bioinformatics the understanding of different molecular cascades has significantly improved. Several phytochemicals have been reported to modulate the expression of key signaling cascades. Bioflavonoids such as genistein have significant potential to develop efficient treatments for cancer. Interplay between genistein and NF-κB modulates various signaling pathways such as NOTCH and PI3K/Akt/mTOR pathway. Genistein prevents activation of Notch1 and its downstream targets and thus inhibits growth and differentiation in colorectal cancer. Genistein promotes cellular growth inhibition by targeting VEGF/VEGFR pathway. Genistein also prevents growth in different tumors by modulating key signaling pathways such as the Wnt/β-catenin, JAK-STAT and JNK pathway. Deeper knowledge of genistein mediated modulation of various signaling networks is crucial for better therapeutic targeting. Accumulating evidence has helped to explain the role of microRNAs in the regulation of key signaling modulators involved in proliferation and metastasis of different malignant tumors. Interaction between miRNAs and genistein requires further investigation which will probably help to develop more efficient treatment. A plethora of miRNAs are involved in the genistein induced inhibition of various tumors. Combination of miRNAs and genistein together with conventional therapies can be useful for controlling the tumor growth and proliferation. Although this is based on only few clinical trials, it seems that NPs loaded with genistein and miRNAs might be an efficient and relatively safe strategy for treatment of different malignant tumors, particularly breast and prostate cancer. Therefore, it is necessary to perform larger clinical trials to prove clinical usefulness and safety of nano-formulations of genistein and miRNAs and compare them with conventional anti-cancer drugs.

## Data Availability

Yes.

## Abbreviations

Akt: Protein kinase B
CDKIs: Cyclin-Dependent Kinase inhibitors
EGFR: Epidermal growth factor receptor
FOLFOX: Fluorouracil Oxaliplatin
FRP: FTF regulatory protein
GDP: Guanosine diphosphate
GTP: Guanosine triphosphate
HCFC2: Host Cell Factor C2
IL: Interleukin
JAK: Janus Kinase
LPR: Low density lipoprotein receptor-related protein
MCM: Minichromosome maintenance protein complex
MDM2: Mouse double minute 2 homolog
MMP: Matrix metalloproteinases
NF-κB: Nuclear factor kappa-light-chain-enhancer of activated B cells
PTEN: Phosphatase and tensin homolog
RTK: Receptor tyrosine kinase
SHOC: Leucine-rich repeat protein
SLPs: Solid-lipid-particulate system
SOX: SRY-box transcription factor
STAT: Signal transducer and activator of transcription
VEGFR: Vascular endothelial growth factor receptor
WIF: Wnt inhibitory factor
