# Correction: Genistein as a regulator of signaling pathways and microRNAs in different types of cancers

**DOI:** 10.1186/s12935-022-02667-y

**Published:** 2022-08-13

**Authors:** Zeeshan Javed, Khushbukhat Khan, Jesús Herrera-Bravo, Sajid Naeem, Muhammad Javed Iqbal, Haleema Sadia, Qamar Raza Qadri, Shahid Raza, Asma Irshad, Ali Akbar, Željko Reiner, Ahmed Al-Harrasi, Ahmed Al-Rawahi, Dinara Satmbekova, Monica Butnariu, Iulia Cristina Bagiu, Radu Vasile Bagiu, Javad Sharifi-Rad

**Affiliations:** 1 Office of Research Innovation and Commercialization (ORIC), Lahore Garrison University, Sector-C, DHA Phase-VI, Lahore, Pakistan; 2 Department of Healthcare Biotechnology, Atta-ur-Rahman School of Applied Biosciences (ASAB), National University of Sciences and Technology (NUST), Islamabad, 44000 Pakistan; 3 Departamento de Ciencias BasicasFacultad de Ciencias, Universidad Santo Tomas, Santiago, Chile; 4 Center of Molecular Biology and Pharmacogenetics, Scientific and Technological Bioresource Nucleus, Universidad de La Frontera, 4811230 Temuco, Chile; 5 School of Life Sciences, Lanzhuo University, Lanzhou, 730000 People’s Republic of China; 6 Department of Biotechnology, Faculty of Sciences, University of Sialkot, Sialkot, Pakistan; 7 Department of Biotechnology, BUITEMS, Quetta, Pakistan; 8 Institute of Biochemistry and Biotechnology, University of Veterinary and Animal Sciences, Lahore, Punjab Pakistan; 9 Department of Life Sciences, University of Management Sciences, Lahore, Pakistan; 10 Department of Microbiology, University of Balochistan, Quetta, Pakistan; 11 Department of Internal Medicine, University Hospital Centre Zagreb, School of Medicine, University of Zagreb, Zagreb, Croatia; 12 Natural and Medical Sciences Research Centre, University of Nizwa, Birkat Almouz , Nizwa, 616 Oman; 13 High School of Medicine, Al-Farabi Kazakh National University, Almaty, Kazakhstan; 14 Banat’s University of Agricultural Sciences and Veterinary Medicine “King Michael I of Romania” From Timisoara, Timisoara, Romania; 15 Victor Babes University of Medicine and Pharmacy of Timisoara Discipline of Microbiology, Timisoara, Romania; 16 Multidisciplinary Research Center On Antimicrobial Resistance, Timisoara, Romania; 17 Preventive Medicine Study Center, Timisoara, Romania; 18 Phytochemistry Research Center, Shahid Beheshti University of Medical Sciences, Tehran, Iran

## Correction: Cancer Cell Int (2021) 21:388 https://doi.org/10.1186/s12935-021-02091-8

The authors would like to clarify that reference [9], which has been retracted, was included in this review article [1] in error. We apologise to readers for any inconvenience caused.
